# The association of clozapine treatment periods with incidence of exposure to assault and head injuries - a cohort study of electronic health records

**DOI:** 10.3389/fpsyt.2025.1704073

**Published:** 2026-01-21

**Authors:** Francesca Kingston, Robert Stewart, Risha Govind, Amelia Jewell, Vishal Bhavsar, James H. MacCabe

**Affiliations:** 1 Maudsley Biomedical Research Centre (BRC), South London and Maudsley National Health Service (NHS) Foundation Trust, London, United Kingdom; 2 Department of Psychological Medicine, Institute of Psychiatry, Psychology and Neuroscience, King’s College London, London, United Kingdom; 3 Section of Women’s Mental Health, The David Goldberg Centre for Health Services and Population Research, Institute of Psychiatry, Psychology and Neuroscience, King’s College London, London, United Kingdom; 4 Department of Psychosis Studies, Institute of Psychiatry, Psychology and Neuroscience, King’s College London, London, United Kingdom

**Keywords:** antipsychotic, clozapine, cohort, head injury, schizophrenia, victimization

## Abstract

People with schizophrenia experience violent victimization, and interventions to reduce this risk are needed. We tested the association of clozapine treatment with risk of exposure to assault and head injuries using electronic health records linked to a national clozapine treatment register and to national hospitalization data. The unadjusted incidence rate ratio (IRR) for clozapine treatment on exposure to assault and head injuries was 0.44 (95% confidence interval [CI]: 0.28-0.69). This effect remained statistically significant after covariate adjustment (0.46 (0.29-0.74); 0.40 (0.22-0.71) in men; 0.64 (0.29-1.43) in women. Clozapine treatment was thus associated with a substantially reduced risk of exposure to assault and head injuries.

## 1 Introduction

There is increased research attention toward patient safety in severe mental illness (SMI), considering the prevalence of victimization, its impacts, and its absence from routine psychiatric assessment (1). Victimization is associated with more severe symptomatology, lack of social support, and repeat victimization (1).

Clozapine is an atypical antipsychotic associated with reduced symptoms and hospitalization in schizophrenia (2). Despite this, clozapine is considered under-prescribed, due to concerns surrounding rare health risks. A study using national registers found clozapine was associated with the lowest relative risk of any crime of all antipsychotics (3). A mirror-image study from our group showed that clozapine initiation is associated with reduced violent crime in the following 2 years compared to the previous 2 years, and this effect is stronger for clozapine than olanzapine (4). However, despite research examining the association of clozapine with perpetration of violence, there is limited research on the association of clozapine treatment with the risk of being subject to violent victimization. Therefore, we tested the association between clozapine treatment and exposure to assaults, traumatic brain injuries, and head injuries in an electronic health records database for a large NHS mental health trust in Southeast London, linked to a register of clozapine treatment and national hospitalization data.

## 2 Methods

### 2.1 Data sources

We analyzed de-identified patient data from the South London and Maudsley (SLaM) NHS Trust, which is made available for secondary analysis via the Clinical Record Interactive Search (CRIS) system (5). CRIS has ethical approval for use in secondary analysis (5) from Research Ethics Committee, Oxford C (reference 23/SC/0257). Patients in CRIS were linked to the Zaponex Treatment Access System (ZTAS), the provider of mandatory blood monitoring for clozapine-treatment SLaM patients, as part of previous work (6). In brief, clozapine treatment periods (with start and end dates) were estimated from full blood count test date information held within ZTAS. We excluded patients with missing age and primary diagnosis data.

### 2.2 Measurements

Clozapine treatment periods were measured by operationalizing start dates as falling when a patient received their first clozapine blood test, or when the blood test was labelled as a baseline measurement, or when the blood test was conducted after an interval of between 35 and 60 days from the previous blood test, based on an algorithm developed in previous clozapine research by a member of the team (6). Clozapine treatment end dates were operationalized as falling when a patient received their final test, or when the test was associated with a red result (i.e., white blood cell count < 3.0 x 10^9^/L and/or neutrophil count < 1.5 x 10^9^/L), or when the treatment status accompanying the test was set to ‘discontinued’, ‘interrupted’, or ‘transferred’. Off-treatment periods were defined as the times between consecutive treatment periods. Dates of exposure to assault and head injury were collected using hospital episode statistics data (7), linked to CRIS (8), for admissions to acute medical hospitals where the discharge diagnosis included ICD-10 diagnostic codes for assault (X85-X99, Y00-Y09), head injury (S00-S09), or traumatic brain injury (S02.0, S02.1, S02.3, S02.7-S02.9, S06.0-S06.9, S07.1). Given small cell sizes for individual assault and head injury outcomes, which cannot be reported separately due to restrictions on CRIS data, all outcome categories (assault, TBI, and AHI) were collapsed into one category, referred to as “assaults and head injuries” which is presented in counts.

### 2.3 Analysis

Stata 17 was used for all analyses. To test the research objective, episodes of clozapine treatment were compared with episodes without treatment in zero-inflated Poisson regressions (estimating incidence rate ratios, IRRs), taking account of clustering of treatment episodes within patients using the Stata “cluster” command. Estimates for men and women were estimated by including a multiplicative interaction term in the final model and then calculating fitted estimates by linear combination. Age (grouped for reporting into the bands: < 16, 16 – 24, 25 – 34, 35 – 44, 45 – 54, 55+), gender, ethnicity (White, Black, Asian, Mixed, Other), and marital status (single, married/cohabiting, divorced/separated, widowed) were collected at referral from structured EHR fields. Substance use diagnosis, and any inpatient psychiatric admission, within the first year following referral were measured using structured fields. Information on aggression, and antipsychotic exposure were collected using natural language algorithms applied to the first year of clinical notes data.

## 3 Results

**Table 1** describes patient sociodemographic characteristics and zero-inflated Poisson model estimates accounting for clustering of treatment episodes within patients. There were 1,345 patients in the study. Prevalence of injuries increased with age, from 3.1% in 16–24-year-olds, to 34.3% in those aged 55 and older. The prevalence of assault and head injury events, expressed as counts, during follow up was similar between male and females (11.9% and 10% respectively). White patients had the highest prevalence of assault and head injury incidents during follow up (15%).

**Table 1:** Description of patient data analysed in this study, and model estimates (incidence rate ratios, IRR, with 95% confidence intervals in parentheses) from zero-inflated Poisson models, for association of clozapine treatment with the rate of any assault and head injury, and gender-stratified estimates.

Demographic characteristics			
	N	Any injury during observation (N, %)	
Age at referral			
< 16	32	-	3.1
16 – 24	363	28	7.7
25 – 34	401	40	10
35 – 44	336	30	8.9
45 – 54	146	29	19.9
55+	67	23	34.3
Gender			
Female	480	48	10
Male	865	103	11.9
Ethnicity			
White	565	85	15
Black	354	34	9.6
Asian	102	10	9.8
Mixed	54	-	3.7
Other	36	-	11.1
Missing	0	-	0
Marital status			
Single	138	46	33.3
Married	86	15	17.4
Missing	1121	90	8.0
Substance use diagnosis within first year			
No	1,276	150	11.8
Yes	69	-	1.5
Aggression within first year			
No	671	89	13.3
Yes	674	62	9.2
Olanzapine exposure within first year			
No	775	95	12.3
Yes	570	56	9.8
Number of clozapine treatment episodes			
1	973	113	11.6
2	249	20	8
3	82	10	12.2
4+	41	-	7.3
Any inpatient admission within first year			
No	936	118	12.6
Yes	409	33	8.1
Accumulated observation time for each respective failure definition, dichotomized at the median (‘short’ = observation times below the median, ‘long’ = observation times above the median), both on and off treatment			
Short	673	110	16.3
Long	672	41	6.1
Accumulated observation time for each respective failure definition, dichotomized at the median (‘short’ = observation times below the median, ‘long’ = observation times above the median), treatment periods only			
Short	673	94	14
Long	672	57	8.5
Accumulated observation time for each respective failure definition, dichotomised at the median (‘short’ = observation times below the median, ‘long’ = observation times above the median), off treatment periods only			
Short	673	95	14.1
Long	672	56	8.3
Total	**1,345**	**151**	**11.2**
Model estimates for associations between clozapine treatment and rates of injury			
Overall		IRR	Outcomes/episodes
No adjustments		0.44 (0.28, 0.69)	88/3808
Adding age, gender, ethnicity		0.46 (0.29, 0.74)	79/3170
Adding aggression, antipsychotics exposure, substance use comorbidity, inpatient admission		0.46 (0.29, 0.74)	79/3170
Gender-stratified model estimates		IRR	D/N
No adjustments Men Women		0.39 (0.22, 0.67) 0.58 (0.28, 1.23)	88/3808
Adding age, ethnicity Men Women		0.40(0.23,0.71) 0.64(0.23,0.71)	79/3170
Adding aggression, antipsychotics exposure, substance use comorbidity, inpatient admission Men Women		0.40(0.23,0.71) 0.64(0.29, 1.43)	79/3170

To protect against identification of individual participants, cells containing < 10 subjects have been redacted (with a “–”).

Bold values represent totals.

The unadjusted IRR for the association between clozapine treatment and rates of any assault and head injury was 0.44 (95% confidence interval [CI]: 0.28, 0.69). This effect remained similar in magnitude after adjustment for covariates (IRR = 0.46, 95% CI: 0.29, 0.74). Gender-stratified analyses indicated an adjusted IRR of 0.40 (0.22, 0.71) for men, and 0.64 (0.29, 1.43) for women. We did not find statistical evidence for interaction by gender.

## 4 Discussion

We found a reduced rate of exposure to assault and head injury experienced by patients when they were on clozapine treatment, compared to periods when they were off clozapine treatment. This rate reduction was greater in magnitude for male patients than for female patients, although we did not find statistical evidence for interaction by gender.

To our knowledge, our study is the first to investigate the association between clozapine treatment exposure and the rate of exposure to assault and head injury incidents. We studied a sample of a patients from a large NHS mental health provider, with available information on periods of clozapine treatment taken from linked ZTAS data. Nevertheless, the final sample size was 1345, limiting precision of our estimates. Our measurement of exposure to assault and head injury incidents was based hospitalization data which is a widely used measure of population violence exposure but is likely to be an underestimate of all incidents of violent injury to which patients may have been exposed.

As blood monitoring is a mandatory condition of clozapine treatment, clozapine-prescribed patients can experience greater contact with services, potentially conferring additional therapeutic effects to the group studied. Previous research has not found clinical contact to account for the negative association between clozapine use and mortality, after adjusting models for inpatient and outpatient clinical contact (9).

We used date information from national mandatory blood monitoring system to compute clozapine treatment start and end dates. This may have introduced inaccuracy. We were unable to measure non-clozapine antipsychotic prescribing in the study population, which is likely to have been variable between on and off clozapine periods, potentially introducing bias into our results. Future research should consider the possible impact of antipsychotic prescribing on exposure to violent injury with detail on treatment periods across a range of antipsychotic drugs.

Clozapine is associated with a range of beneficial effects in people diagnosed with schizophrenia. Clozapine has previously shown to be associated with reduced self-harming behavior and lower mortality (10), and reduced perpetration of violence (4). Our findings suggest that clozapine treatment could be associated with reduced exposure to assault and head injury in patients with schizophrenia. Future research should examine this relationship in larger study populations with detailed information on prescribing. This evidence could be relevant to clinical discussions about the risks and benefits of clozapine treatment and guide personalized management of patients with schizophrenia.

## Data Availability

The data analyzed in this study is subject to the following licenses/restrictions: No new data was created in this study. The data that support the findings of this study are not publicly available due to their containing information that could compromise the privacy of research participants. Research material availability is not applicable to this article as no new materials were used in this study. The analytic code supporting the findings of this study is not available to other researchers via a recognized repository, but can be made available on request from author VB. . Requests to access these datasets should be directed to Vishal Bhavsar, vishal.2.bhavsar@kcl.ac.uk.
